# Morphology-Based Evaluation of Pollen Fertility and Storage Characteristics in Male *Actinidia arguta* Germplasm

**DOI:** 10.3390/plants14152366

**Published:** 2025-08-01

**Authors:** Hongyan Qin, Shutian Fan, Ying Zhao, Peilei Xu, Xiuling Chen, Jiaqi Li, Yiming Yang, Yanli Wang, Yue Wang, Changyu Li, Yingxue Liu, Baoxiang Zhang, Wenpeng Lu

**Affiliations:** 1 Institute of Special Animals and Plants Sciences, Chinese Academy of Agricultural Sciences, Changchun 130112, China; qinhongyan@caas.cn (H.Q.); tcsfst@sohu.com (S.F.); zkxpl@126.com (P.X.); lijiaqionly@hotmail.com (J.L.); yym0312@163.com (Y.Y.); wangyanli@caas.cn (Y.W.); wangyue05@caas.cn (Y.W.); lcy_lcy2002@163.com (C.L.); liuyingxue82@163.com (Y.L.); 2 Pomology Institute, Jilin Academy of Agriculture Sciences, Gongzhuling 136100, China; zhaoying8107@163.com; 3 Applied Technology Research and Development Center for Sericulture and Special Local Products of Hebei Universities, Institute of Sericulture, Chengde Medical University, Chengde 067000, China; enchxl@163.com

**Keywords:** hardy kiwiftuit (kiwiberry), pollen grains, germination capacity, pollen morphology

## Abstract

*Actinidia arguta* is a dioecious plant, and the selection of superior male germplasm is crucial for ensuring effective pollination of female cultivars, maximizing their economic traits, and achieving high-quality yields. This study evaluated 30 male germplasms for pollen quantity, germination capacity, storage characteristics, and ultrastructural features. Results revealed significant variation in pollen germination rates (1.56–96.57%) among germplasms, with ‘Lvwang’, ‘TL20083’, and ‘TG06023’ performing best (all >90% germination). The storage characteristics study demonstrated that −80 °C is the optimal temperature for long-term pollen storage in *A. arguta*. Significant variations were observed in storage tolerance among different germplasms. Among them, Lvwang exhibited the best performance, maintaining a germination rate of 97.40% after 12 months of storage at −80 °C with no significant difference from the initial value, followed by TT07063. Pollen morphology was closely correlated with fertility. High-fertility pollen grains typically exhibited standard prolate or ultra-prolate shapes, featuring a tri-lobed polar view and an elliptical equatorial view, with neat germination furrows and clean surfaces. In contrast, low-fertility pollen grains frequently appeared shrunken and deformed, with widened germination furrows and visible exudates. Based on these findings, the following recommendations are proposed: ① Prioritize the use of germplasms with pollen germination rates >80% as pollinizers; ② Establish a rapid screening system based on pollen morphological characteristics. This study provides important scientific basis for both male germplasm selection and efficient cultivation practices in *A. arguta* (kiwiberry).

## 1. Introduction

*Actinidia arguta* Sieb. et Zucc. (commonly known as kiwiberry or hardy kiwi) is a large deciduous vine belonging to the genus *Actinidia* within the family Actinidiaceae. This species is characterized by its distinctive fruit flavor, exceptional nutritional value, remarkable cold hardiness, and disease resistance. These traits make it both a highly valuable small-berry crop and an excellent parental resource for *Actinidia* hybridization breeding. *A. arguta* stands out as one of the most cold-tolerant species within the *Actinidia* genus, with dormancy survival capabilities extending to –30 °C [1]. This remarkable hardiness positions it as a commercially valuable small-berry crop for temperate and cold regions worldwide. Commercial production has expanded significantly since the early 2000s, with established orchards in New Zealand, the United States, Japan, Chile, and multiple European countries [2,3]. Despite China’s abundant wild genetic resources, large-scale commercial cultivation of *A. arguta* began only in 2010. According to available statistics, the total cultivated area in China reached approximately 5533 hectares by 2024 [4], demonstrating rapid industry development despite its relatively recent inception.

As a functionally dioecious species, *A. arguta* necessitates systematic male plant deployment in commercial orchards. The breeding of superior male cultivars is equally important as female cultivar selection. It ensures proper pollination and fertilization of female plants, enabling full expression of their economic traits and achieving high yield and premium fruit quality goals. However, research on the evaluation and breeding of male *A. arguta* genetic resources remains limited. In production, most allocated male plants are either seedlings or individuals with undefined flowering traits, leading to insufficient fertilization of female plants. This subsequently causes flower and fruit abscission, significantly reducing both fruit yield and quality. Among various floral characteristics used for male germplasm evaluation, flower quantity and pollen fertility serve as key quality indicators, with pollen fertility being the crucial factor directly affecting pollination, fruit set rate, and fruit size in female plants [5,6]. Preliminary investigations revealed significant variation in pollen germination rates (ranging from >90% to nearly 0%) among different male *A. arguta* germplasm under identical *in vitro* culture conditions, consistent with findings by wang [7].

In practical production, kiwifruit often suffers from insufficient natural pollination due to asynchronous flowering phenology between male and female flowers and unfavorable environmental conditions, leading to abnormal fruit development (e.g., misshapen fruits and reduced fruit weight). Artificial supplementary pollination serves as a key solution to this issue, with its effectiveness largely dependent on the maintenance of pollen viability. Since the interval between pollen collection and application may range from several hours to months, establishing an effective pollen storage system is crucial [8]. Studies have shown that pollen from most plant species can maintain viability for several years under low-temperature conditions ranging from −10 °C to −200 °C [9]. For *Actinidia* species, Bomben et al. [10] and Abreu and Oliveira [11] demonstrated that pollen can be effectively preserved for 54–160 weeks through temperature regulation. Notably, Naik et al. [12] demonstrated that kiwifruit pollen cryopreserved at −200 °C for 12 months maintained a 100% fruit set rate post-storage. However, systematic research on pollen storage characteristics in *A. arguta* remains relatively scarce, and further investigation is still needed regarding its optimal storage conditions and the storage stability variations among different male germplasms.

This study systematically evaluated 30 male *A. arguta* germplasm resources for key reproductive traits including flowering characteristics, pollen production, and germination rates, with the objective of identifying elite male genotypes with superior comprehensive traits. Through scanning electron microscopy (SEM) analysis, we conducted a thorough examination of pollen micromorphological features and investigated their correlations with pollen fertility. The findings provide both theoretical foundations for male cultivar breeding and practical guidance for optimal pollinizer selection in commercial orchards, offering significant potential for enhancing *A. arguta* yield and fruit quality.

## 2. Materials and Methods

### 2.1. Plant Materials

A total of 30 male germplasm accessions of *A. arguta* were collected from the National Woody Plant Germplasm Repository of *Actinidia arguta* of the Institute of Special Animal and Plant Sciences of the Chinese Academy of Agricultural Sciences, Zuojia Town, Jilin City, Jilin Province, China (44°00′ N; 126°01′ E). The tested germplasms were 10–20 years old, cultivated using a T-trellis system, and managed under standard agricultural practices.

### 2.2. Experimental Procedures

#### 2.2.1. Flower Quantity Assessment and Collection

**Figure 1:**
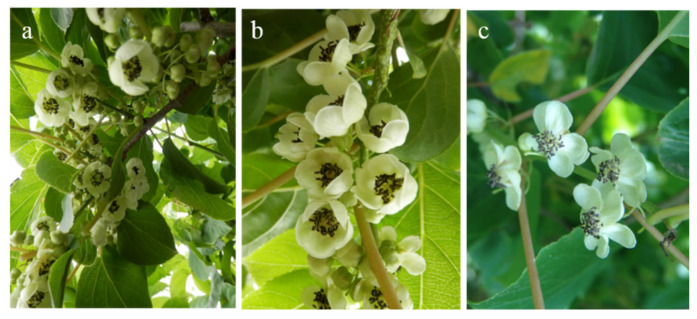
Evaluation of flower abundance in male *Actinidia arguta* germplasm. (**a**) High flower abundance, scores of 3. (**b**) Medium flower abundance, scores of 2. (**c**) Low flower abundance, scores of 1. Photographed on 15 June 2022.

At the initial full bloom stage (when 15% of flowers had opened, On 13 June 2022), the evaluation of flowering characteristics was conducted for the 30 male germplasms, accompanied by sample collection. According to the visual evaluation criteria for flowering characteristics shown in Figure 1, the flower abundance was categorized into three levels: high (more than 50% of inflorescences had 6–7 flowers), medium (more than 50% of inflorescences had 4–5 flowers), and low (more than 50% of inflorescences had 1–3 flowers). Scores of 3, 2, and 1 were assigned to high, medium, and low abundance, respectively. For each germplasm resource, approximately 1000 large flower buds were collected at the pre-flowering stage. Anthers were carefully extracted using forceps, placed in sulfuric acid paper bags, and dried over silica gel at 25 ± 2 °C for 12 h to allow natural dehiscence and pollen release. The pollen was then filtered through 100–200 mesh sieves to remove anther debris and other impurities, yielding purified pollen for subsequent experiments.

#### 2.2.2. Anther Number and Pollen Quantity Measurement

Closed large flower buds were selected for anther counting. Ten flowers constituted one replicate (three replicates total) to calculate the mean anther number per flower. For pollen quantification, 100 non-dehisced anthers were placed in centrifuge tubes and oven-dried at 35 °C. A 5 mL aliquot of 20% (*w*/*v*) sodium hexametaphosphate solution was added to the centrifuge tube, followed by thorough vortex mixing (≥30 s at 2000 rpm) to achieve homogeneous pollen suspension. A 0.5 mL aliquot was diluted to 5 mL, from which 1 µL was sampled for microscopic examination using an OLYMPUS SHE biological microscope (Olympus Corporation, Shinjuku, Tokyo, Japan). Pollen grains within the field of view were counted to determine the mean pollen count per anther. This procedure was repeated in triplicate.

#### 2.2.3. Pollen Germination Rate Assay

Pollen germination was evaluated using in vitro culture. Pollen grains were evenly distributed on solid medium (0.01% boric acid + 0.03% calcium nitrate + 10% sucrose + 1% agar, pH 5.8) at an optimal density of 50–100 grains/mm^2^, which was predetermined to avoid overcrowding while ensuring sufficient sample size for statistical analysis. Petri dish lids were lined with filter paper to absorb excess moisture, followed by incubation at 20 °C in darkness for 5 h. Germination rates were determined by examining ≥50 pollen grains per field across 10 randomly selected microscopic fields (three replicates). Germination rate (%) = (germinated pollen grains/total pollen grains) × 100%.

#### 2.2.4. Pollen Storage at Low and Ultra-Low Temperatures

Pollen from five genotypes (‘Lvwang’, ‘TL20083’, ‘TG09032’, ‘TG01081’, and ‘TG06023’) dried with silica gel for 12 h, was subjected to low-temperature or ultra-low temperature storage testing. The pollen samples of each germplasm were aliquoted into 2 mL sterile centrifuge tubes (0.5× *g* per tube), sealed, and stored in ziplock bags. The samples were preserved under three different temperature conditions: (1) 4 °C (laboratory refrigerator, ±1 °C fluctuation); (2) −20 °C (laboratory freezer, ±1 °C fluctuation); and (3) −80 °C (ultra-low temperature freezer, ±1 °C fluctuation). All samples were stored in the dark. The initial germination rate of the pollen samples was measured before the experiment. After storage periods of 2 months and 12 months, the samples were retrieved and thawed at 25 ± 2 °C, followed by germination rate assessment according to Section 2.2.3. Thawed samples were exclusively used for immediate testing and were not subjected to re-freezing for subsequent analyses. Three replicates were performed for each treatment.

#### 2.2.5. Scanning Electron Microscopy (SEM)

Desiccated pollen from all 30 male germplasms was mounted on stubs, gold-coated via ion sputtering, and imaged using an XL30 Feg ESEM system (FEI Company, Hillsboro, OR, USA). Morphological characterization was conducted, including comparative analysis of pollen features across all 30 germplasm accessions. Quantitative measurements of key morphological parameters (polar axis length, equatorial axis length, aperture length, and aperture width) were obtained using ImageJ software 1.x (National Institutes of Health, Bethesda, MD, USA) with scale calibration.

#### 2.2.6. Pollen Morphological Abnormality Analysis

**Figure 2:**
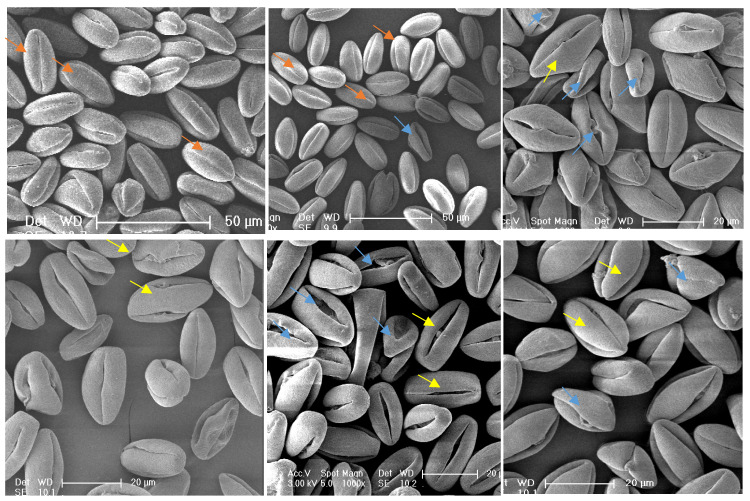
Comparative analysis of pollen morphological characteristics in male *A. arguta* germplasms reveals distinct ultrastructural differences between normal and abnormal grains under scanning electron microscopy (SEM). Normal pollen (indicated by orange arrows) exhibit intact prolate morphology with neatly defined apertures and clean exine surfaces, while moderately abnormal pollen (yellow arrows) display slight aperture deformation or minimal widening. Severely abnormal pollen (blue arrows) demonstrate pronounced morphological aberrations including collapsed structures, disorganized aperture patterns, and visible surface exudates.

Pollen morphology was classified as normal or aberrant (Figure 2 criteria) under SEM. Abnormal and slightly abnormal rates were quantified to evaluate morphological anomalies across germplasms.

#### 2.2.7. Statistical Analysis

Analyses were performed using Excel and SAS 9.1 statistical software (SAS Institute Inc., Cary, NC, USA). For significance testing of differences among different treatments for the same index, one-way analysis of variance (ANOVA) was performed followed by least significant difference (*LSD*) post hoc multiple comparisons. Correlation analysis between different indices was conducted using Pearson’s correlation coefficient. Data visualization was performed with SIGMA Plot 14.0 software (Systat Software Inc., San Jose, CA, USA).

## 3. Results

### 3.1. Floral Characteristics and Pollen Germination Capacity of Different Germplasms

As shown in Table 1, significant variations were observed among male germplasms in flower abundance, mean anther number per flower, and pollen quantity per anther. The evaluation identified: 20 germplasms with high flower abundance, 3 with medium abundance, 8 with low abundance. Anther number ranged from 36.0 to 64.5 per flower, with most germplasms (40–48). Three accessions (TT05022, TG01081, TG06081) exceeded 50 anthers/flower, while six showed <40. Pollen production varied from 12,875 to 42,250 grains/anther, TT08093 and TG06063 were the most productive.

Pollen germination rates varied significantly among male germplasms (1.56–96.57%). The highest germination rates were observed in Lvwang (96.57%), TL20083 (95.85%), TG06023 (93.47%) and TG01081 (87.84%). In contrast, TT04043 (1.56%), TL17061 (2.63%), TT07031 (3.18%), and TT03021 (4.33%) showed the lowest rates. These results demonstrate substantial genetic diversity in pollen viability among *A. arguta* male germplasms, providing a scientific basis for selecting superior pollinizers.

### 3.2. Pollen Storage at Low and Ultra-Low Temperatures

**Figure 3:**
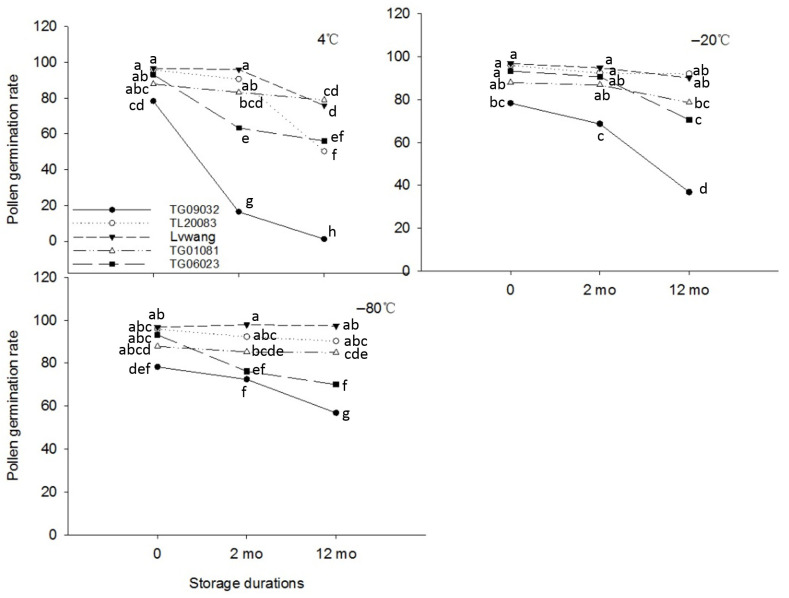
Pollen germination of different *A. arguta* germplasm accessions under different temperature storage conditions over varying durations. Different superscript lowercase letters denote statistically significant differences among treatments (*p* < 0.05), analyzed using the Least Significant Difference (LSD) multiple comparison method.

**Figure 4:**
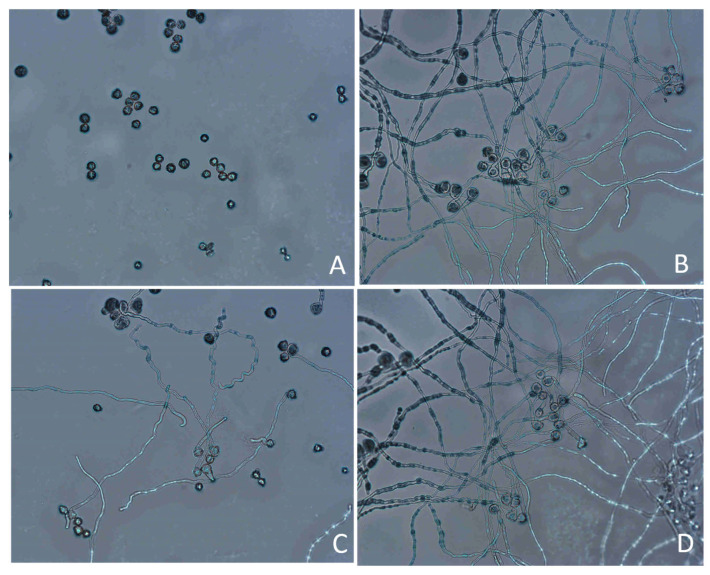
Pollen germination of *A. arguta* under different storage conditions. (**A**): ‘TG09032’ pollen failed to germinate after 12 months at 4 °C. (**B**): ‘Lvwang’ pollen germinated after 12 months at −20 °C. (**C**): ‘TG09032’ pollen showed significantly reduced germination after 12 months at −20 °C. (**D**): ‘Lvwang’ pollen maintained high germination after 12 months at −80 °C.

Figure 3 illustrates the pollen germination performance of five male *A. arguta* germplasm accessions under different storage temperatures (−80 °C, −20 °C, and 4 °C) across varying time periods (0, 2, and 12 months). Pre-storage assessments revealed that all tested germplasms exhibited pollen germination rates exceeding 78.60%, with Lvwang, TL20083, and TG06023 demonstrating exceptional performance by achieving rates above 90% (Figure 3). Pollen viability progressively declined across all storage temperatures over time. After 12 months at 4 °C, all germplasms except TG01081 (which showed only an 8.86% reduction) experienced significant viability losses ranging from 20.64% to 77.50%, with TG09032 displaying the most pronounced decline (77.50%) to a final germination rate of 1.11% (Figure 4A). In contrast, −20 °C storage resulted in comparatively smaller reductions, with Lvwang maintaining 90.11% germination (Figure 4B) after 12 months while TG09032 decreased by 41.72% (Figure 4C). Pollen storage at −80 °C was most effective, with *Lvwang* retaining 97.40% germination (Figure 4D), showing no significant difference from initial values and TG09032 maintaining 56.84% viability, significantly higher than that under other temperature treatments (*p* < 0.05). Comprehensive analysis confirmed −80 °C as the optimal storage condition, with the five germplasms ranked by 12-month germination performance as Lvwang > TL20083 > TG01081 > TG06023 > TG09032.

### 3.3. Pollen Morphological Characteristics of Different Germplasms

The morphological characteristics of pollen observed under a scanning electron microscope are shown in Table 2. The results indicate that pollen grains from various male *A. arguta* germplasms predominantly exhibited prolate shapes, displaying tri-lobed polar views and elliptical equatorial views. The pollen grain dimensions ranged from (21.56–33.80) μm × (10.27–19.84) μm. The germination pores types were primarily tricolporoidate (80.0%), with fewer tricolpate forms (20.0%), and some grains showed visible exudates from germination furrow. The germination pores measured 17.04–30.10 μm in length with neat edges, spaced 0.97–8.14 μm apart, and showed a polar/equatorial axis ratio of 1.80–2.21. Additionally, the pollen surfaces exhibited distinct wrinkled-blocky ornamentation, with significant variation in pattern depth among different germplasms.

**Figure 5:**
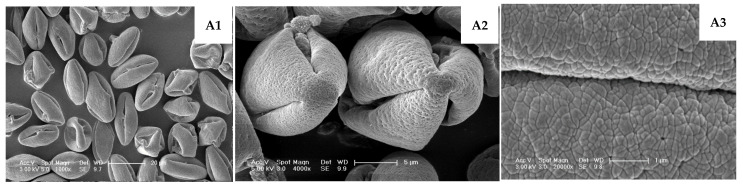

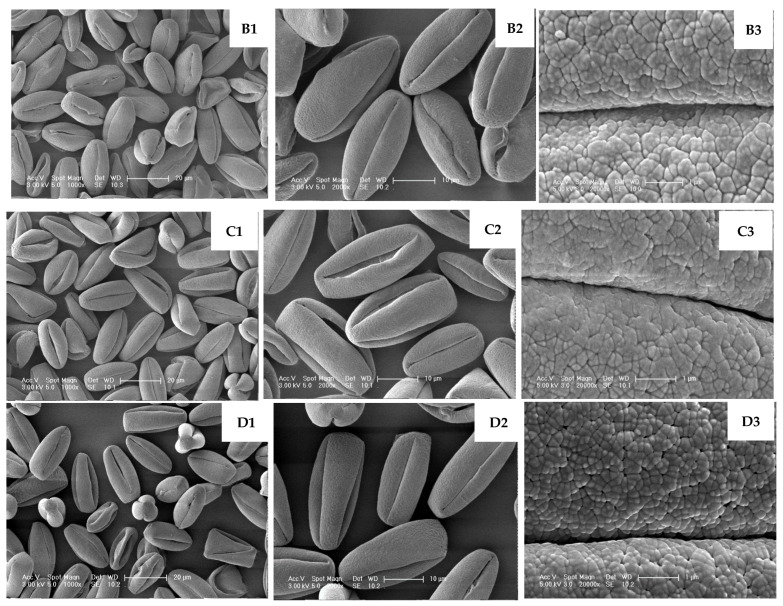

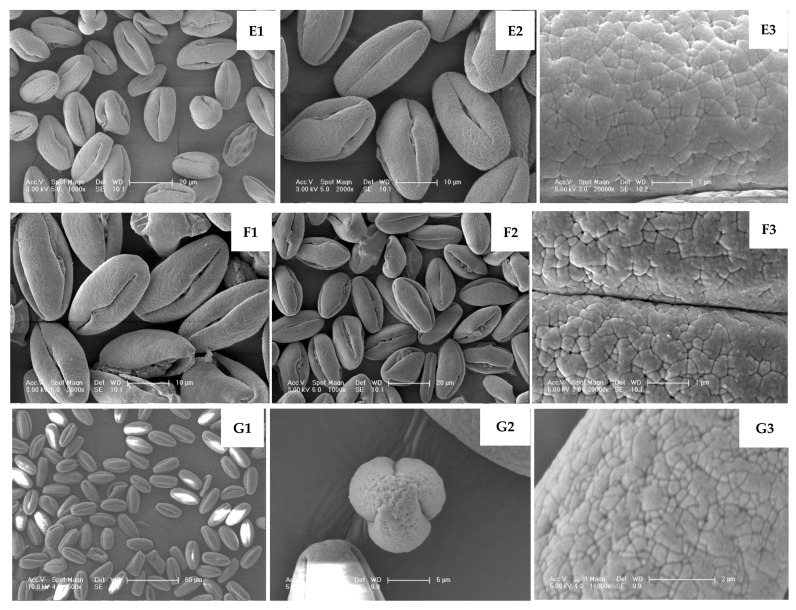

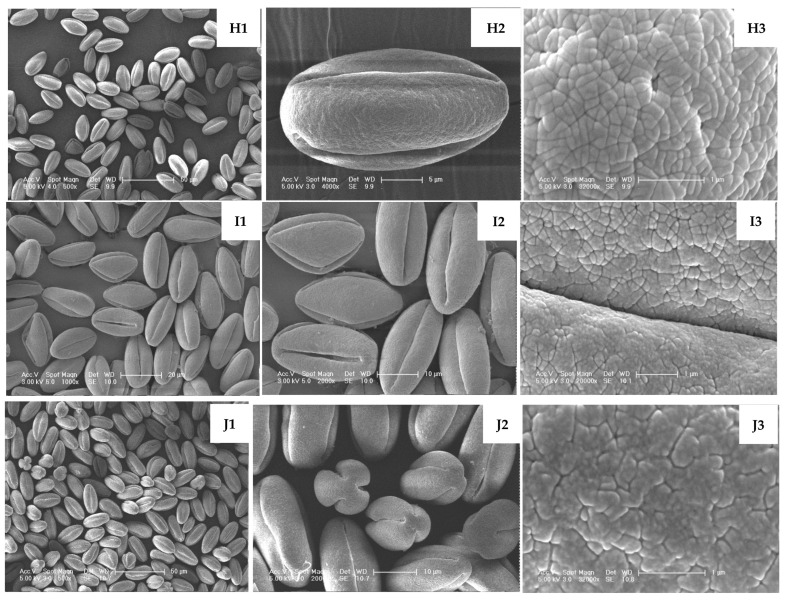
Scanning electron microscopy (SEM) observations of pollen morphology in male *A. arguta* germplasms. TT03021: (**A1**–**A3**) Prolate, very few normal, most grains shrunken/deformed with widened apertures and visible exudates; TT04043: (**B1**–**B3**) Hyperprolate, few apertures fissured, no exudates; TT07031: (**C1**–**C3**) Prolate, few apertures fissured, no exudates; TT07063: (**D1**–**D3**) Hyperprolate, very few shrunken; TL17093: (**E1**–**E3**) Prolate, most deformed–shrunken/shortened, few apertures with exudates; TL12043: (**F1**–**F3**) Prolate, multiple fissured apertures with exudates; TL20063: (**G1**–**G3**) Prolate, relatively uniform, occasional deformations; Lvwang: (**H1**–**H3**) Prolate, uniform; TG01081: (**I1**–**I3**) Hyperprolate, relatively uniform; TG09032: (**J1**–**J3**) Hyperprolate, Exceptionally uniform.

Significant morphological differences were observed in pollen grains among male germplasms. Lvwang and TG09032 exhibited highly uniform pollen morphology, characterized by typical prolate shapes with plump grains, uniform aperture widths, and absence of exudates. TG01081 pollen showed relative consistency, predominantly prolate but including some grains with shortened polar axes appearing as irregular prolate forms with uneven aperture widths.

In contrast, TT03021 displayed remarkable abnormalities, with only a small minority maintaining prolate shapes while most grains appeared shrunken and deformed, exhibiting significantly widened apertures accompanied by exudates. Although TT04043 pollen was mainly prolate, partial grains were shrunken with distorted apertures and minor exudates. While TT07031 produced plump, prolate pollen grains, they exhibited significantly widened germination apertures without exudates. TT07063 showed approximately 50% deformed grains, including shrunken forms and those lacking distinct polarity. TL17093 pollen was predominantly deformed with partial shrinkage and visible exudates at aperture sites. Although TL12043 grains maintained plumpness, all displayed distorted apertures with consistent exudation. These morphological variations provide critical microstructural criteria for assessing pollen fertility and selecting elite male germplasms in *A. arguta* breeding programs.

As shown in Table 3, correlation analysis of 12 traits revealed that flower abundance, mean anther number, and pollen quantity per anther showed no significant correlation with pollen germination rate or morphological characteristics. However, pollen germination rate was significantly positively correlated with normal pollen percentage, while exhibiting significant negative correlations with abnormal and slightly abnormal pollen percentages. Furthermore, normal pollen percentage was significantly negatively correlated with abnormal pollen percentage, slightly abnormal pollen percentage, equatorial axis length, and aperture spacing, but positively correlated with pollen germination rate and P/E ratio (polar/equatorial axis ratio). These results demonstrate intrinsic links between pollen morphology and fertility, providing critical criteria for selecting high-fertility male germplasms.

## 4. Discussion

As a functionally dioecious species, *A. arguta* necessitates deliberate male plant deployment in commercial orchards to ensure reproductive success. The breeding of superior male cultivars holds equal importance to female cultivar development in ensuring effective pollination and fertilization, optimal expression of commercially valuable traits, and the production of high-yielding, premium-quality fruits. Among the various floral characteristics used to evaluate male germplasm resources, flower abundance and pollen fertility emerge as the most critical selection criteria. Investigations of 30 male accessions demonstrated significant variability in flowering capacity, anther number per flower, and pollen production per anther. For commercial cultivation, germplasms exhibiting both profuse flowering and abundant pollen output should be prioritized for propagation, as these traits serve as reliable secondary indicators for identifying elite male genetic resources.

Beyond floral abundance and pollen quantity, pollen fertility emerges as the key determinant of pollination efficiency, fruit set rate, and final fruit size in female plants. Pollen viability and germination capacity are fundamental to successful pollination, which directly governs fruit quality and yield in *A. arguta* cultivars [13]. This parameter is especially critical for hybrid breeding programs and artificial pollination protocols. Various methods are available for assessing pollen fertility, including in vivo and in vitro germination assays, histochemical staining, and fertilization capability tests [14,15,16,17,18]. Among these, in vitro germination assays have gained widespread adoption due to their simplicity, efficiency, and reliable diagnostic value. By tracking pollen tube growth dynamics, this method provides accurate insights into germination potential and fertility levels [15,16,19,20,21,22].

Our study employed in vitro germination to evaluate male *A. arguta* germplasms, revealing striking variability in pollen germination rates (1.56–96.57%). Elite germplasms demonstrated >90% germination, whereas low-fertility accessions showed <10% viability, with some nearly complete sterility. Similarly, wang et al. [7] also found that the pollen activity of different kiwifruit cultivars (strains) varied significantly. Among the 41 samples, 87.8% of the samples had pollen activity between 40% and 80%, while the lowest was only 5.01%. The use of male germplasm with underdeveloped pollen or low germination rates for pollination significantly reduces fruit quality and yield, highlighting the importance of breeding and promoting male varieties with high pollen fertility for commercial production. Notably, some male germplasm exhibits normal stamen morphology and sufficient pollen production, yet their pollen germination rates remain extremely low. It is indirectly speculated that this phenomenon may be related to abnormal pollen structural development, providing important clues for further research into the mechanisms of pollen development.

Pollen storage stability and fertility are key criteria for evaluating male germplasm resources, particularly for ensuring successful artificial pollination and addressing asynchronous flowering issues in production systems. Research indicates that pollen longevity is primarily governed by genetic factors, but is also significantly influenced by environmental conditions, especially storage temperature [23]. This study compared the pollen preservation efficacy of five germplasms under three storage temperatures (4 °C, −20 °C, and −80 °C). The results demonstrated that pollen stored at −80 °C exhibited the smallest decline in germination rate after 12 months, significantly outperforming storage at 4 °C and −20 °C. This observation aligns with the advantages of ultra-low temperature storage (−80 °C) reported by Li et al. [24]. Additionally, Naik et al. [12] found that kiwifruit pollen stored at −200 °C for 12 months maintained a 100% fruit set rate, whereas pollen stored at room temperature and 4 °C failed to set fruit. In our study, even under identical −80 °C storage conditions, significant variations in viability retention were observed among different germplasms. The pollen storage stability ranking was ‘Lvwang’ > ‘TL20083’ > ‘TG01081’ > ‘TG06023’ > ‘TG09032’, confirming the decisive role of genetic factors in storage tolerance. For commercial production, we recommend adopting −80 °C ultra-low temperature storage technology and prioritizing pollen resources with high storage stability, as this approach effectively maintains pollen viability and ensures reliable artificial pollination quality.

Pollen morphological characteristics serve as crucial indicators for plant phylogenetic studies and taxonomic identification [25,26,27]. The ultrastructural features observed under scanning electron microscopy (SEM) play a definitive role in species differentiation [17,21,28]. This study revealed that *A. arguta* pollen exhibits typical prolate shapes (polar axis/equatorial axis ratio = 1.8–2.21) with predominantly tricolporate apertures (80%), consistent with the fundamental morphological characteristics of the *Actinidia* genus [29] and matching descriptions of *A. arguta* by Stasiak et al. [30]. However, these findings contrast with reports describing sub-spherical pollen with triporate features (P/E ratio = 1.37), suggesting that such morphological variations may result from different environmental conditions at collection sites, indicating potential geographical variation or phenotypic plasticity in *Actinidia* pollen morphology.

Significant progress has been made in understanding pollen abortion mechanisms, particularly the relationship between pollen sterility and microstructural abnormalities, which has been extensively studied in various plants including *Gleditsia* [31], *Vitis* [32], and *Jasminum* [33]. Early studies [34,35] also confirmed significant correlations between pollen fertility and morphological indices in *citrus* species. Our SEM observations of different *A. arguta* germplasms revealed substantial variations in pollen morphology. High-fertility germplasms produced uniform, typically prolate pollen grains with trilobed polar views and elliptical equatorial views. These grains appeared plump with uniform apertures and clean surfaces devoid of exudates. In contrast, low-fertility pollen predominantly produced shriveled and deformed grains with conspicuously wide apertures and prevalent exudates. All pollen grains displayed distinctive, regular exine ornamentation, though the depth of these patterns varied markedly among germplasms. Correlation analysis demonstrated significant relationships between pollen germination rates and morphological characteristics. These results suggest that low germination rates may directly correlate with abnormal pollen structures, potentially caused by genetic factors in the plants, though further verification is required. This demonstrates that pollen appearing normal macroscopically may exhibit abnormal microstructure. Conversely, pollen fertility might be indirectly inferred through microscopic structural examination. These findings establish a reliable foundation for rapid fertility assessment through morphological evaluation and develop a morphology-based screening method for selecting superior male *A. arguta* germplasms, providing important practical applications for enhancing breeding efficiency.

## 5. Conclusions

Significant variation in pollen germination rates (1.56–96.57%) was observed among different male *A. arguta* germplasms, with practical recommendation to prioritize cultivars or elite lines exhibiting >80% germination as pollinizers to ensure high yield and superior fruit quality. Ultra-low temperature storage at −80 °C enables long-term pollen preservation, although substantial inter-germplasm variation in storage tolerance was observed. A strong correlation exists between pollen fertility and morphological characteristics: high-fertility germplasms produce uniform, standard prolate grains with tri-lobed polar views, regular elliptical equatorial views, well-filled structures, neat apertures of consistent width, and clean surfaces devoid of exudates; whereas low-fertility pollen predominantly displays deformed/shrunken grains with aberrant apertures and exudates. The established rapid screening method based on pollen morphology provides a reliable and efficient technical approach for identifying elite germplasms, significantly enhancing breeding efficiency.

## Figures and Tables

**Table 1:** Flowering capacity and pollen germination rates of different male germplasms.

Germplasm ID	Visual Evaluation of Flower Abundance	Mean Anther Number	Pollen Grains per Anther	Pollen Germination Rate (%)
TT03021	2	40.2 hijk	27,610 bcd	4.33 k
TT04043	1	42.4 fghij	16,190 ijkl	1.56 k
TT05022	1	64.2 a	16,625 ijkl	17.62 j
TT06051	1	43.6 efghi	24,445 cdefg	35.21 gh
TT06023	3	44.6 defgh	18,645 fghijkl	26.44 i
TT07031	2	39.0 ijk	14,375 jkl	3.18 k
TT07033	1	36.0 k	13,190 kl	41.44 fg
TT07063	1	37.4 jk	20,875 defghij	7.13 k
TT08093	3	39.2 ijk	42,250 a	37.80 gh
TL21013	3	44.6 defgh	20,250 efghijk	45.58 f
TL20083	1	36.2 k	28,065 bc	95.85 a
TL18072	3	44.6 defgh	25,875 bcde	32.85 hi
TL17093	1	41.0 ghijk	26,375 bcde	18.04 j
Lvwang	3	47.4 bcdef	16,750 ijkl	96.57 a
TL17061	3	42.2 ghij	16,690 ijkl	2.63 k
TL15012	1	41.0 ghijk	12,875 l	19.25 j
TL15022	3	40.0 hijk	16,190 ijkl	36.03 gh
TL14101	3	45.4 cdefg	16,815 hijkl	54.99 e
TL13091	3	47.6 bcde	20,815 defghij	63.90 e
TL12043	3	48.4 bcde	24,315 cdefg	17.65 j
TG02023	2	45.8 cdefg	17,355 ghijkl	65.98 d
TG01081	3	45.4 cdefg	26,645 bcde	87.84 b
TG04021	3	51.0 b	22,500 cdefghi	47.32 f
TG06011	3	39.8 hijk	23,930 cdefgh	58.25 e
TG06023	3	48.6 bcde	20,750 defghij	93.47 ab
TG06063	3	49.4 c	32,315 b	73.05 cd
TG06081	3	46.0 bcdefg	14,750 jkl	34.30 gh
TG07053	3	50.0 bc	18,565 fghijkl	37.66 gh
TG07031	3	44.0 efghi	24,690 cdef	40.56 fg
TG09032	3	48.4 bcde	18,375 fghijkl	78.48 c

Note: In the visual evaluation of flower abundance, numerical ratings represent: 1: Low flower abundance, 2: Medium flower abundance, 3: High flower abundance. Values followed by different lowercase letters within a column indicate statistically significant differences (*p* < 0.05), as determined by the Least Significant Difference (LSD) method for multiple comparisons.

**Table 2:** Principal morphological characteristics of pollen from male *A. arguta* germplasms.

Germplasm ID	Morphological Description	Pollen Size (Polar Axis × Equatorial Axis)/μm	P/E Ratio	Aperture Type	Aperture Length/μm	Aperture Width/μm	Abnormal Percentage/%	Slightly Abnormal Percentage/%	Figure
TT03021	Prolate; very few normal; most grains shrunken/deformed with widened apertures and visible exudates	(25.46–30.67) × (13.48–16.21)	1.85	Tricolporoidate	21.17–25.95	1.05–3.99	16.85 cdefg	72.28 ab	Figure 5A1–A3
TT04043	Hyperprolate; few shrunken grains; apertures with exudates	(25.51–30.59) × (11.98–15.96)	2.06	Tricolporoidate (with exudate)	22.29–28.07	1.81–2.94	10.09 efghijk	18.56 mno	Figure 5B1–B3
TT05022	Prolate; few apertures fissured	(23.52–27.97) × (12.76–14.73)	1.88	Tricolporate	19.22–26.06	1.19–3.48	15.7 cdefgh	38.15 ghijk	
TT06051	Hyperprolate; few slightly deformed	(25.27–31.78) × (11.70–16.11)	2.19	Tricolporoidate	23.94–29.51	0.90–2.28	7.61 hijkl	45.43 efghi	
TT06023	Hyperprolate; few slightly deformed; few apertures fissured	(25.89–33.80) × (12.04–16.14)	2.15	Tricolporoidate	22.65–30.10	1.73–3.04	8.11 ghijkl	34.69 ghijk	
TT07031	Prolate; few apertures fissured; no exudates	(24.85–30.17) × (13.08–19.60)	1.93	Tricolporoidate	21.32–26.87	1.86–4.35	8.39 fghijkl	30.83 ijklm	Figure 5C1–C3
TT07033	Hyperprolate; most apertures fissured; few deformed with exudates	(25.02–30.37) × (12.57–16.03)	2.03	Tricolporate	21.00–27.09	2.39–4.25	17.74 cde	35.24 ghijk	
TT07063	Hyperprolate; very few shrunken	(22.19–30.92) × (10.27–13.78)	2.21	Tricolporoidate	18.54–27.40	1.18–2.27	15.48 cdefghi	34.13 hijkl	Figure 5D1–D3
TT08093	Prolate; most apertures fissured; few with exudates	(25.36–29.38) × (11.88–14.96)	1.93	Tricolporate (with exudate)	20.62–24.59	1.66–4.89	17.25 cde	75.92 a	
TL21013	Prolate; few shortened and slightly deformed	(25.50–29.31) × (12.63–17.16)	1.90	Tricolporoidate	22.69–26.54	0.97–3.23	7.5 hijkl	44.15 efghi	
TL20083	Prolate; relatively uniform; occasional deformations	(21.56–28.76) × (2.38–15.08)	1.97	Tricolporoidate	18.47–25.76	1.14–3.79	0.71 l	12.38 nop	Figure 5G1–G3
TL18072	Prolate; few apertures fissured	(22.79–30.12) × (12.30–15.15)	1.97	Tricolporoidate	21.08–28.24	1.61–5.10	20.81 bc	42.53 fghij	
TL17093	Prolate; most deformed–shrunken/shortened; few apertures with exudates	(22.99–30.48) × (12.97–16.46)	1.80	Tricolporoidate (with exudate)	17.04–26.08	1.51–4.10	17.89 cde	54.31 cdef	Figure 5E1–E3
Lvwang	Prolate; uniform	(26.40–28.11) × (12.89–19.84)	1.90	Tricolporoidate	22.09–25.36	1.76–3.51	3.06 jkl	0.00 p	Figure 5H1–H3
TL17061	Prolate; few deformed–shrunken/triangular-pyramidal	(23.72–27.53) × (11.57–16.34)	1.87	Tricolporoidate	20.21–25.33	2.39–5.33	19.15 bcd	40.12 fghijk	
TL15012	Prolate; most apertures fissured; irregular surface	(23.24–30.54) × (13.19–16.19)	1.81	Tricolporate, Tricolporoidate	20.91–26.31	2.23–6.05	17.09 cdef	61.83 abcd	
TL15022	Prolate; few apertures fissured	(25.23–29.75) × (12.57–15.42)	1.99	Tricolporoidate	18.88–27.29	2.03–3.92	3.59 jkl	58.52 bcde	
TL14101	Prolate; most apertures fissured and twisted	(23.92–29.88) × (13.25–17.47)	1.82	Tricolporate, Tricolporoidate	18.70–26.35	2.07–8.14	27.02 b	63.05 abc	
TL13091	Prolate; relatively uniform; occasional shrunken grains	(26.75–28.43) × (12.82–18.02)	1.95	Tricolporoidate	22.91–27.70	2.06–5.26	20.21 bcd	46.99 defgj	
TL12043	Prolate; multiple fissured apertures with exudates	(26.47–29.96) × (14.21–17.82)	1.81	Tricolporate (with exudate)	20.27–27.71	3.53–6.19	40.55 a	49.4 cdefg	Figure 5F1–F3
TG02023	Hyperprolate; relatively uniform	(27.37–29.27) × (12.56–15.12)	2.07	Tricolporoidate	21.61–25.91	1.40–2.65	0.63 l	7.88 op	
TG01081	Hyperprolate; relatively uniform	(25.42–28.29) × (11.93–14.29)	2.04	Tricolporoidate	22.62–26.40	1.24–2.72	0.88 l	6.29 op	Figure 5I1–I3
TG04021	Hyperprolate; very few normal; most grains shrunken/deformed with widened apertures and visible exudates	(24.00–29.17) × (10.75–14.54)	2.16	Tricolporoidate	19.21–25.63	1.81–4.36	7.45 hijkl	43.83 efghi	
TG06011	Prolate; Generally uniform; Occasional deformed grains	(26.08–28.73) × (12.14–16.50)	1.90	Tricolporoidate	21.64–26.24	1.47–3.77	6.74 ijkl	19.37 lmno	
TG06023	Hyperprolate; Minority slightly deformed	(25.05–29.03) × (12.08–14.30)	2.01	Tricolporoidate	22.79–26.99	1.64–2.86	2.87 kl	25.89 klmn	
TG06063	Prolate; Relatively uniform; Minority slightly deformed	(23.72–28.07) × (11.55–14.39)	1.99	Tricolporoidate	21.54–26.15	1.08–1.93	0.93 l	2.69 p	
TG06081	Prolate; Minority with fissured apertures; Pollen deformation present	(23.78–30.32) × (13.75–16.66)	1.83	Tricolporoidate	21.48–27.64	2.36–5.25	11.62 defghijk	31.28 ijklm	
TG07053	Hyperprolate; 50% deformed grains (partial shrinkage or triangular-pyramidal transformation)	(23.42–28.96) × (11.59–14.51)	2.01	Tricolporoidate	19.65–27.28	1.62–3.36	11.65 cdefghi	28.64 jklm	
TG07030	Hyperprolate; Isolated deformed grains	(26.27–30.92) × (11.95–14.81)	2.12	Tricolporoidate	20.33–28.58	1.25–4.17	5.35 jkl	40.73 fghijk	
TG09032	Hyperprolate; Exceptionally uniform	(27.47–30.32) × (13.03–15.12)	2.03	Tricolporoidate	22.73–27.32	1.71–3.42	0 l	9.04 op	Figure 5J1–J3

Note: Values followed by different lowercase letters in a column differ significantly (*p* < 0.05), analyzed using the Least Significant Difference (LSD) multiple comparison method.

**Table 3:** Correlation analysis among different parameters.

	Visual Flower Abundance Rating	Mean Anther Number per Flower	Pollen Grains per Anther	Pollen Germination Rate	Normal Pollen Percentage	Abnormal Pollen Percentage	Slightly Abnormal Pollen Percentage	Polar Axis Length	Equatorial Axis Length	Aperture Length	Aperture Spacing	Polar/Equatorial Axis Ratio (P/E)
Visual Flower Abundance Rating	1											
Mean Anther Number per Flower	0.25	1										
Pollen Grains per Anther	0.17	−0.12	1									
Pollen Germination Rate	0.35	0.16	0.18	1								
Normal Pollen Percentage	0.06	0.14	−0.12	0.62 **	1							
Abnormal Pollen Percentage	−0.06	0.01	0.00	−0.55 **	−0.82 **	1						
Slightly Abnormal Pollen Percentage	−0.06	−0.18	0.16	−0.58 **	−0.96 **	0.63 **	1					
Polar Axis Length	−0.03	−0.23	−0.24	−0.14	0.02	−0.08	0.01	1				
Equatorial Axis Length	0.08	−0.10	−0.22	−0.25	−0.44 *	0.49 **	0.37 *	0.13	1			
Aperture Length	0.11	0.04	−0.27	0.05	0.30	−0.31	−0.27	0.73 **	0.03	1		
Aperture Spacing	0.18	−0.07	−0.17	−0.30	−0.65 **	0.77 **	0.52 **	−0.04	0.65 **	−0.19	1	
Polar/Equatorial Axis Ratio (P/E)	−0.09	−0.05	0.05	0.12	0.40 *	−0.48 **	−0.31	0.47 **	−0.81 **	0.40 *	−0.61 **	1

Note: ** indicates significant difference at the 0.01 level; * indicates significant difference at the 0.05 level.

## Data Availability

The data generated in this study were freely available to any researcher.
